# Diagnostic Capabilities of Islet Autoantibodies in Children with New-Onset Type 1 Diabetes Mellitus and Healthy Siblings

**DOI:** 10.17691/stm2020.12.6.04

**Published:** 2020-12-28

**Authors:** K.G. Korneva, L.G. Strongin, E.V. Kolbasina, M.V. Budylina, N.V. Makeeva, V.E. Zagainov

**Affiliations:** Associate Professor, Department of Endocrinology and Internal Medicine; Privolzhsky Research Medical University, 10/1 Minin and Pozharsky Square, Nizhny Novgorod, 603005, Russia;; Professor, Head of the Department of Endocrinology and Internal Medicine; Privolzhsky Research Medical University, 10/1 Minin and Pozharsky Square, Nizhny Novgorod, 603005, Russia;; Pediatric Endocrinologist, Head of the Department of Endocrinology; Nizhny Novgorod Regional Children’s Clinical Hospital, 211 Vaneeva St., Nizhny Novgorod, 603136, Russia;; Head of the Department of Pediatric Endocrinology and Gastroenterology; Republican Children’s Clinical Hospital of the Ministry of Health of the Chuvash Republic, 27 Fedora Gladkova St., Cheboksary, 428020, Russia;; Pediatric Endocrinologist, Chief Non-Staff Pediatric Endocrinologist; Children’s Republican Clinical Hospital, 10 Meditsinskaya St., Yoshkar-Ola, the Republic of Mari El, 424005, Russia; Associate Professor, Head of the Department of Faculty Surgery and Transplantology Privolzhsky Research Medical University, 10/1 Minin and Pozharsky Square, Nizhny Novgorod, 603005, Russia;

**Keywords:** type 1 diabetes mellitus, islet autoantibodies, siblings, immunological diagnosis of T1DM

## Abstract

**The aim of the study** is to determine the diagnostic utility of several islet autoantibodies and their combinations in order to identify individuals susceptible to type 1 diabetes mellitus (T1DM) among healthy siblings in the pediatric population within the scope of the development of a screening program.

**Materials and Methods.:**

A total of 424 children were evaluated, 260 children with new-onset T1DM and 164 healthy children with brothers and/or sisters with T1DM.

Blood tests for a complex of autoantibodies to insulin (IAA), tyrosine phosphatase (IA-2A), zinc transporter 8 (ZnT8A), pancreatic β-cells (ICA), and glutamate decarboxylase (GADA) were conducted in all the subjects with the enzyme immunoassay method.

**Results.:**

It was found that the diagnostic utility of individual autoantibodies is not equal and varies with age. The optimal age groups for the immunological control of the risks of developing type 1 diabetes in healthy siblings were determined. The highest risks were noted with the combination of GADA, ZnT8A, and IA-2A.

**Conclusion.:**

Islet autoantibodies may serve as prognostic markers of the risk of developing type 1 diabetes in healthy siblings.

## Introduction

The occurrence of autoantibodies (AAb) to islet cell antigens is an established sign of the development of an autoimmune response directed against insulin-producing β-cells and characterizes the onset of the preclinical stage of type 1 diabetes mellitus (T1DM). Currently, five main types of AAb are used as markers of autoimmune inflammation: to insulin (IAA), islet cells (ICA), glutamic acid decarboxylase 65 (GADA), tyrosine phosphatase (IA-2A), and zinc transporter 8 (ZnT8A) [1].

The test results for AAb showed their ambiguity. The risk of developing T1DM has been established to grow with an increase in the number of detected AAb types. Though, the AAb presence shows instability. In the course of the dynamic follow-up, they can disappear, particularly in case of the presence of a single positive AAb type [2]. The composition of AAb types can also vary without apparent regularity and the titers do not always increase by the time of T1DM manifestation which complicates their prognostic interpretation [3]. Besides, there are age and ethnic features of the presence of certain AAb.

Within the scope of practical implementation of the scientific idea of creating monoclonal antibodies to prevent the development of T1DM, a screening program on the identification of patients at the preclinical stage of the development of T1DM who have a sufficient supply of functioning islets and, accordingly, the potential for further immunotherapy is under development. The assessment of the predictive capabilities of the known AAb is one of the possible ways to identify the target group. In spite of a large number of studies devoted to studying AAb in T1DM, the diagnostic utility of identifying particular AAb at various stages of the development of T1DM, the choice of the target group, the optimal age, and follow-up time still present great scientific and practical interest.

**The aim of the study** is to determine the diagnostic utility of identifying particular islet autoantibodies and their combinations in order to detect individuals susceptible to T1DM among healthy siblings in the pediatric population within the scope of the development of the screening program.

## Materials and Methods

424 children were evaluated in the Nizhny Novgorod region, Chuvashia, and the Republic of Mari El, of which 260 children with new-onset T1DM (group 1) and 164 healthy children having brothers and/or sisters with T1DM (group 2). The groups did not differ statistically significantly in terms of age and gender. The median age of the children was 8 [4.5; 11] years in group 1 and 8 [4; 15] years in group 2. The ratio of boys and girls was 57.7 and 42.3% in the group with T1DM, 58.5 and 41.5% in the group without T1DM, respectively.

The study was conducted in accordance with the Declaration of Helsinki (2013) and approved by the Ethics Committee of the Privolzhsky Research Medical University (Nizhny Novgorod, Russia). Informed consent was obtained from the patients’ parents.

All patients with a new-onset T1DM and healthy siblings underwent a blood test for AAb, glycated hemoglobin (HbA1c), and C-peptide. The test systems for enzyme-linked immunosorbent assay were used in the Tecan Sunrise absorbance microplate reader (Austria GmbH, Austria): IgG antibodies to insulin — IAA (Orgentec, Germany), tyrosine phosphatase — IA-2A (Medipan, Germany), zinc transporter 8 — ZnT8A (Medipan), β-cells of the pancreas — ICA (Biomerica, USA), glutamate decarboxylase — GADA (Euroimmun AG, Germany). The AAb values higher than the reference ones were considered positive. The HbA1c test was performed by high-performance liquid chromatography in the VARIANT IV TURBO analyzer (Bio-RAD, USA/France), the C-peptide test — by solid-phase chemiluminescence immunoassay in the IMMULITE 2000XPi analyzer (Siemens Healthcare Diagnostics, USA).

**The statistical data** were processed using the Statistica 12.0 software. The quantitative values are presented as median and interquartile range [Q1; Q3], the discrete data — in the form of feature frequencies (in percent). The distribution of signs did not correspond to the law of normal distribution according to the Shapiro–Wilk test. The comparison analysis of medians in the independent samples was performed using the nonparametric Mann–Whitney test and the Kruskal–Wallis test. The statistical analysis of the frequency distribution was carried out using contingency tables and the χ^2^ test with Yates’s correction for continuity. The quantitative relationship between the features was analysed by the Spearman’s rank correlation method. The differences were considered statistically significant at a significance level of p<0.05.

## Results

The median age at onset of T1DM was 8 [4.5; 11] years with an increase in morbidity in the age range from 2 to 12 years (74) in comparison with 12% of the cases of under 2 years and 14% after 12 years (p<0.001).

The comparative characteristics of the incidence of several positive AAb in the groups are presented in Table 1. The overwhelming majority of the children with new-onset T1DM were positive for GADA, followed by IA-2A and ZnT8A with the same incidence. In the group of healthy AAb-positive children, GADA prevailed, ICA and IA-2A were registered slightly less frequently. The groups did not statistically differ in the ICA incidence.

**Table 1:** Incidence of single AAb in the groups (%)

AAb type	Group 1 (n=260)	Group 2 (n=164)
GADA	89.2 (n=232)	17.1* (n=28)
ICA	16.3 (n=42)	15.3 (n=25)
IAA	7.7 (n=20)	1.8** (n=3)
IA-2A	73.1 (n=190)	14.6* (n=24)
ZnT8A	69.6 (n=181)	6.7* (n=11)

Note. statistically significant differences when comparing the groups: * p<0.0001; ** p=0.009.

In the group of healthy children, the majority of children (88.4%) were AAb-negative or only one AAb type was noted. In almost half of the cases with T1DM manifestation, a combination of three AAb types was encountered. 3.8% of the cases were AAb-negative (Table 2).

**Table 2:** The total number of AAb types in the patients in the groups (%)

Total of ААb number types	Group 1 (n=260)	Group 2 (n=164)
0	3.8 (n=10)	60.4 (n=99)
1	13.9 (n=36)	28 (n=46)
2	21.2 (n=55)	7.3 (n=12)
3	45 (n=117)	4.3 (n=7)
4	15.7 (n=41)	0
5	0.4 (n=1)	0

Note. statistically significant differences when comparing the groups, p<0.0001.

**Figure 1:**
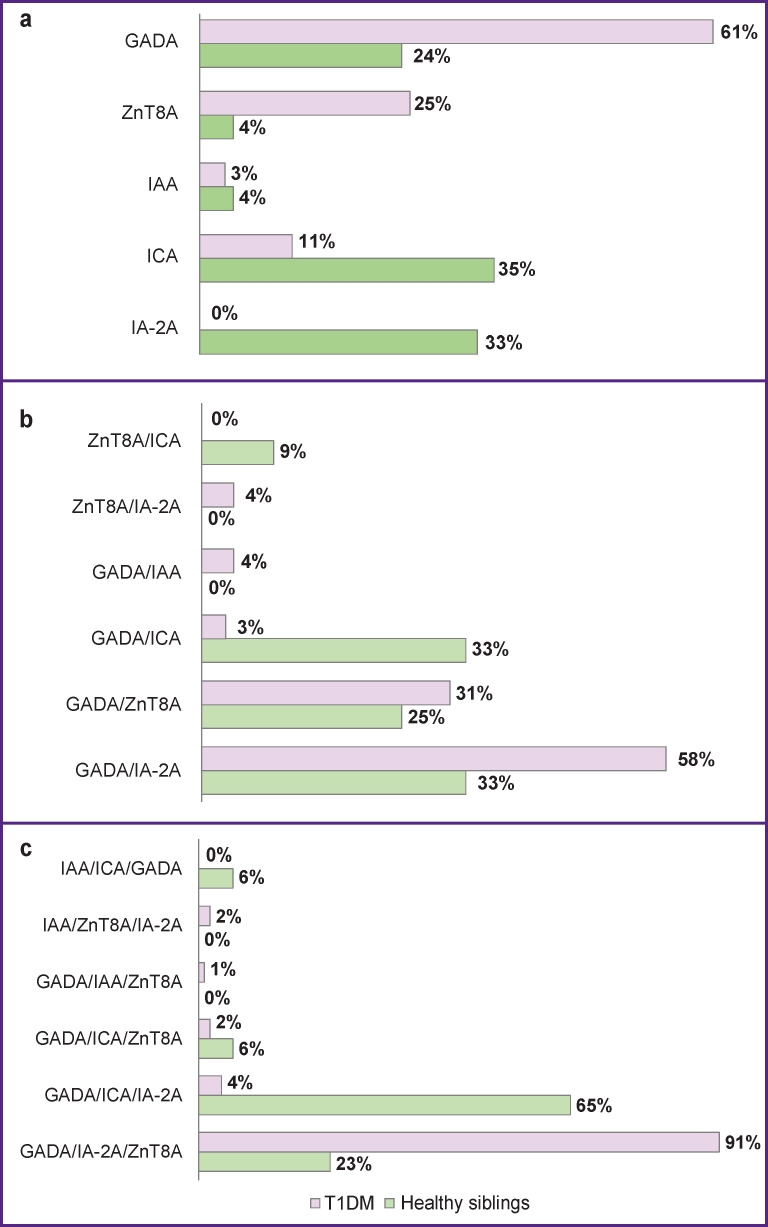
Distribution of AAb among the patients seropositive for multiple autoantibodies: (a) one at a time; (b) two; (c) three

The incidence of single AAb, as well as their combinations in the children with new-onset T1DM and healthy ones, is presented in Figure 1. The children with T1DM and with a single AAb type were positive for GADA in 61% and ZnT8A in 25% of the cases. Interestingly, no IA-2A-positives were noted in this group. Positive correlations were found between the titer levels of GADA/IA-2A (R=0.472; p<0.0001), GADA/ZnT8A (R=0.212; p<0.0005), and IA-2A/ZnT8A (R=0.389; p<0.0001). The most frequent combination of positive AAb was formed by the pairs of GADA/IA-2A and IA-2A/ZnT8A (p<0.001). In 91% of the children with new-onset T1DM, a combination of GADA/IA-2A/ZnT8A was recorded.

In the group of healthy children, such combinations were found with a lower frequency: GADA/IA-2A — in 33%, GADA/ZnT8A — in 25%. There was a positive relationship between the IA-2A/ZnT8A titers (R=0.192; p<0.014). One child each was positive for a combination of GADA/ICA/IA-2A, GADA/ICA/IAA, or GADA/ICA/ZnT8A and 4 children were positive for a GADA-IA-2A/ZnT8A combination. 2 children with the latter combination manifested T1DM after 6 months.

**Figure 2:**
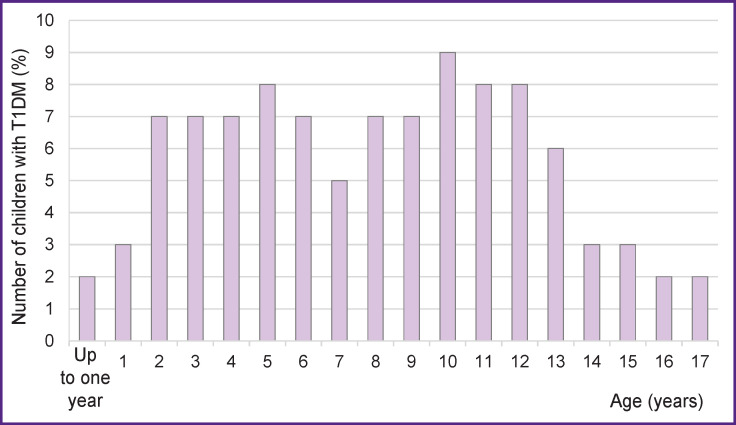
The incidence of new-onset age-dependant T1DM

The age of the T1DM onset (Figure 2) correlated positively with the ZnT8A titer (R=0.126; p=0.043) and negatively with the IAA titer (R=–0.226; p=0.0003). The median age in the ZnT8A-positive children was 8 [5; 11] years in comparison with 7 [3; 11] years in the ZnT8A-negative children (p=0.044). The IAA positives, in turn, were recorded at a younger age. The median age with the IAA-positive results was 3 [2.5; 5] years in comparison with 8 [5; 11] years in the IAA-negative individuals (p=0.0003). The dominant AAb in the children under 2 years old were GADA (83%) and IA-2A (42%). ZnT8A, IAA, and ICA accounted for 30, 17, and 17%, respectively.

The analysis of the subgroups by age of T1DM manifestation demonstrated that the incidence of the IAA-positives recorded in the preschool period (0–6 years — 15%) is statistically significantly greater in comparison with that detected in primary school (7–12 years — 3%) and adolescence (13 years and older — 3%) (p=0.001). For the rest of AAb, no significant differences were found. In the group of healthy siblings, no regularities were found.

The younger children with T1DM showed higher IAA titers. The ZnT8A titer tended to increase at the age of 7–12 years with a further decrease. In the group of healthy children, the titer of any AAb was significantly lower, with the exception of the trend towards an increase in the IAA titer at a younger age (Table 3).

**Table 3:** AAb titer in the age subgroups of the studied children (Me [Q1; Q3])

Age	ААb type			
	GADA	IAA	IA-2A	ZnT8A
***In children with T1DM***				
Reference range	0–4 IU/ml	0–10 U/ml	0–8 U/ml	0–15 IU/ml
0–6 years (n=195)	140.8 [33.0; 331.9]	3.3 [2.0; 6.3]	111.3 [7.5; 445.0]	138.7 [9.5; 453.8]
7–12 years (n=117)	127.5 [30.4; 285.7]	2.0 [1.1; 3.0]	191.5 [7.9; 566.0]	293.8 [13.4; 464.3]
>13 years (n=38)	145.0 [62.4; 436.3]	1.9 [0.9; 2.5]*	485.8 [7.5; 591.0]	94.1 [13.1; 508.6]**
***In children without T1DM***				
Reference range	0–4 IU/ml	0–10 U/ml	0–8 U/ml	0–15 IU/ml
0–6 years (n=195)	1.4 [1.2; 1.8]	2.0 [0.9; 3.0]	6.1 [5.1; 7.1]	5.5 [4.3; 7.4]
7–12 years (n=117)	1.4 [1.2; 2.5]	2.0 [1.4; 2.8]	5.8 [5.0; 7.0]	5.3 [4.0; 6.8]
>13 years (n=38)	1.5 [1.3; 2.1]	1.6 [1.0; 2.3]^+^	6.2 [5.2; 7.1]	5.4 [4.0; 7.4]

Note. statistically significant differences when comparing the groups: * p<0.001; ** p=0.08 (in the T1DM group); ^+^ p=0.07 (in the group without T1DM).

**Figure 3:**
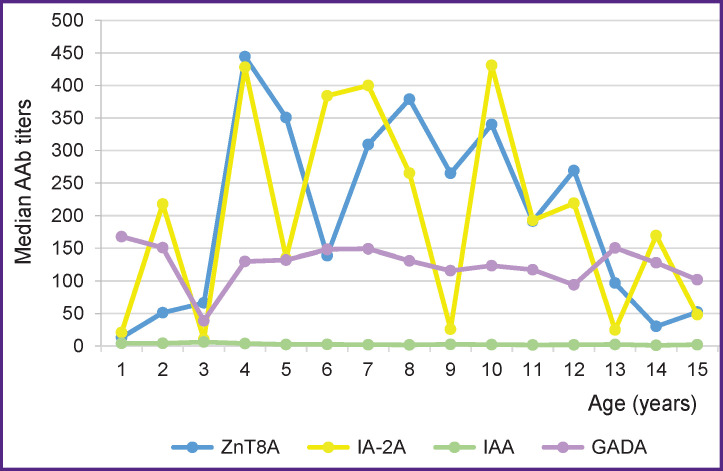
Median AAb titers depending on the age of T1DM manifestation

The AAb titer analysis dependent on the age of disease manifestation (Figure 3) showed that the medians of GADA and IAA titers had steadily stable values in all age groups, the GADA titer ones slightly decreasing at the age of 3 years. The median titers for IA-2A and ZnT8A differed in general by unidirectional sawtooth fluctuations, the trend of which increased after the age of two years and decreased after 12 years.

During the follow-up, 4 boys out of the healthy siblings manifested T1DM. The data are available for two children. Patient M. progressed to T1DM at the age of 8 years, just like his brother had done earlier. Patient R. progressed to T1DM at the age of 2 years, his brother had fallen ill earlier, at the age of 1 year (Table 4). In both of the children, an increase in the level of glycated hemoglobin and a decrease in the level of C-peptide were detected 5 months before the onset of the T1DM clinical symptoms. Initially, there was an increase in three types of AAb: GADA, IA-2A, ZnT8A, the titers of which significantly decreased during T1DM manifestation, with the exception of ZnT8A in patient R. He was also found to be ICA positive.

**Table 4:** AAb titers in the patients progressing to T1DM during the follow-up

ААb	Patient M.		Patient R.	
	Initial titer	After 5 months	Initial titer	After 5 months
GADA (IU/ml)	527.50	140.50	729.05	89.17
IAA (U/ml)	1.56	2.75	2.65	2.35
IA-2A (U/ml)	501.70	404.0	2276.0	181.56
ZnT8A (IU/ml)	3623.0	473.0	212.09	171.60
ICA	0	0	0	1.41
HbA1c (%)	9.0	9.1	5.9	8.4
С-peptide (ng/ml)	0.39	0.31	0.52	0.37

## Discussion

Currently, the risk of developing T1DM is believed to be influenced by age, as well as the amount, type, and combination of AAb.

This risk has been noted [4, 5] to be inversely proportional to age. Our data demonstrate that T1DM is manifested more often at the age of 2 to 12 years. This can determine the target age group for immunological monitoring of healthy siblings.

The presence of multiple AAb is known to signal a high risk of T1DM manifestation. As a rule, 70% of children have three or four positive AAb types by the time the clinical symptoms of diabetes appear, and only 10% have at least one positive AAb type [6]. In our work, almost half of the sick children were positive for 3 types of AAb and approximately the same number of children had two and four AAb types each. The concept of multiple AAb includes a persistent detection of two or more positive types of AAb [2], therefore, it is advisable to evaluate and follow up the children who are seropositive for at least two types of AAb as individuals from a high-risk group for T1DM development. Single positive AAb usually disappear within two years after seroconversion [2].

However, even among individuals with multiple AAb, the risk of developing T1DM is different. The previous studies [7, 8] have shown that early onset and rapid seroconversion over time to several AAb are associated with a high risk of disease progression. In addition, the type of AAb combination is also important.

The critical period for the onset of an islet autoimmune response occurs before the age of 8 years, although earlier and later onset is not excluded either. It is believed that IAA begin to be detected at an early age — from 1 to 4 years, reaching a peak by 8 years with a subsequent sharp decline. GADA tend to be registered later, after two years, and the maximum potential for the risk of disease progression develops by 14 years [4]. It has been shown that the risk of developing T1DM decreases with a decrease in the IAA titer, regardless of the status of other AAb [8]. Our data indicate an increase in both the titer and IAA incidence at a young age in T1DM patients. In the group of healthy siblings, a tendency to increased IAA titers was observed in the younger age group. ZnT8A titers were highest in the group of children in whom diabetes was manifested later, at the age of 7–12 years, which is consistent with other works [9]. The detection of single IA-2A also correlated with a low risk, increasing with the appearance of GADA [10]. Among the children with T1DM, the incidence of single AAb was higher in GADA-positive individuals, followed by ZnT8A and ICA.

The combination of different AAb also carries different risks. ZnT8A and IA-2A appear closer to the clinical onset of the disease. In individuals initially positive for ZnT8A/IA-2A, T1DM progresses to the clinical stage faster, regardless of the type of other joined AAb, for example, for those initially positive for a combination of IAA/GADA. The predictive risks of single IAA and GADA are low, but when they are combined, the risk may increase. Therefore, the joint determination of these AAb is more prognostically significant than their separate determination [10–12]. It has been established that the identification of three positive AAb, two of which necessarily being ZnT8A and IA-2A, represents the subclinical course of T1DM. At the same time, the progression of T1DM may slow down with the loss of positive IAA [5].

We have shown that the GADA/ZnT8A and GADA/ IA-2A pairs are detected in the vast majority of the new-onset T1DM patients and in more than half of the cases in the group of healthy siblings. An example of this is the data on AAb in two healthy siblings who progressed to T1DM during the follow-up. The combination of high IAA/ IA-2A titers increases the risk of developing T1DM within five years after the appearance of the first AAb [13].

Most researchers who have studied the predictive utility of ZnT8A have concluded that their determination facilitates risk stratification in individuals with other positive AAb [14].

The predictive utility of ICA is not high in comparison with that of other islet AAb. The presence of ICA can carry additional risks for the children who already have other AAb. However, according to our data, 11% of the children with new-onset T1DM had only single ICA.

The AAb titers can also be predictors of disease progression. The risk of multiple AAb was significantly increased in the presence of an increased GADA titer, and the risk of dysglycemia was increased with an increase in the IA-2A titer [10]. In our case, no significant regularities in the AAb titer were found. Median GADA and IAA titers were consistently elevated in all age groups. The medians of IA-2A and ZnT8A titers showed unidirectional fluctuations within the increased values.

## Conclusion

The results of the study showed that the screening and dynamic follow-up of the children with a risk of developing T1DM (in healthy siblings) aged 2 to 12 years old are most effective in the individuals with two or more positive AAb types. It is advisable to conduct the dynamic follow-up of the children with single AAb within 1–2 years after their appearance. The most significant markers of the preclinical stage of T1DM are GADA, IA-2A, and ZnT8A. GADA may be informative in any age group. IA-2A and ZnT8A appear, as a rule, later (with the progression of the disease) and can provide additional information, especially in case of positive GADA. IAA positives are common in younger children.

The results obtained do not contradict, but are not totally in line with the data of other studies. This may depend on ethnic differences in the subjects, the time of the identification of detectable AAb at the preclinical stage, or their combination. Undoubtedly, AAb detection provides valuable, though not comprehensive, information. Considering the undoubted impact of genetic susceptibility on the development of T1DM in combination with a number of environmental factors, additional information on AAb properties will help to make a model of a patient at risk of developing T1DM in the future. The accumulation of knowledge on early prognosis of T1DM may ultimately enable a successful intervention to prevent the disease in general.
